# The use of a communication tool about diet at the child health centre: A cluster randomized controlled trial

**DOI:** 10.1002/nop2.498

**Published:** 2020-04-13

**Authors:** Bettina Holmberg Fagerlund, Sølvi Helseth, Lene F. Andersen, Milada C. Småstuen, Kari Glavin

**Affiliations:** ^1^ Department of Nursing and Health Promotion Faculty of Health Sciences OsloMet – Oslo Metropolitan University Oslo Norway; ^2^ Department of Nutrition Institute of Basic Medical Sciences Faculty of Medicine University of Oslo Oslo Norway; ^3^ Department of Health VID Specialized University Oslo Norway

**Keywords:** child, child health services, clinical trial, counselling, food, nursing, nutrition, preschool child, public health nursing, vegetables

## Abstract

**Aim:**

To investigate the effect of a communication tool about diet used in public health nurse consultations with parents compared with standard consultations concerning the 2‐year‐old child's diet.

**Design:**

A cluster randomized controlled trial.

**Methods:**

Ten municipalities were selected randomly and matched in pairs. In each pair, the control or intervention group was randomly allocated. Parents were recruited to participate from January 2015 to January 2017. In intervention clusters, a communication tool about diet was used to help the parents (*N* = 140) to focus on a healthy diet for their child. In the control clusters, parents (*N* = 110) attended standard consultations. The participants completed semi‐quantitative food frequency questionnaires at baseline and end point.

**Results:**

No effect of the intervention was seen on the child's daily intake of vegetables or saturated fat, or body mass index. Significantly fewer parents desired more information about food for toddlers in the intervention than in the control group.

## 1 INTRODUCTION

According to Statistics Norway (2016), almost all parents and their under‐school‐aged children in Norway use child health centres (CHCs) providing extensive, widely available preventive health care in the municipalities on a voluntary basis and free of charge (Norwegian Directorate of Health, 2017). Aspects of food and feeding practices are central to the counselling schedule of public health nurses (PHNs) at the CHC (Norwegian Directorate of Health, 2016, 2017). This reflects parents’ search for trustworthy information if they have concerns and queries related to their children's food and feeding practices (Holmberg Fagerlund, Helseth, Andersen, Småstuen, & Glavin, 2018). It is essential for parents to have a good understanding of their child's nutritional requirements because young children are totally dependent on their parents making decisions for them (Hobbie, Baker, & Bayerl, 2000).

## 2 BACKGROUND

Public health nurses often find it complicated to deal with nutrition and counselling about children's diet in their everyday practice (Holmberg Fagerlund, Pettersen, Terragni, & Glavin, 2016; Ilmonen, Isolauri, & Laitinen, 2012; Magnusson, Kjellgren, & Winkvist, 2012). Counselling based on a one‐sided cognitive approach towards eating appears to have limited impact on healthy food choices among children. A review indicated that deriving pleasure from eating healthy foods or from contextual cues generated by parental attitudes to food and feeding might encourage children to adopt a balanced diet in the long term (Marty, Chambaron, Nicklaus, & Monnery‐Patris, 2018). An association between healthy dietary habits in children and parents having higher education has been shown in several studies (Luque et al., 2018; Rasmussen et al., 2006; Vepsäläinen et al., 2018). A questionnaire study among parents (*N* = 234) of 1‐ to 5‐year‐old children revealed that short‐duration breastfeeding or the food neophobia in the child was associated with a risk of poor dietary patterns in children later. No associations were found between the dietary patterns of children and the age when solid foods had been introduced (Bell, Jansen, Mallan, Magarey, & Daniels, 2018). A longitudinal study among children (*N* = 633) in five European countries by Luque et al. (2018) indicated that educational interventions should focus not only on the introduction of positively weighted foods, but also on avoidance of discretionary low‐quality foods at early ages. Dietary patterns, particularly between 1 and 2 years, persisted into mid‐childhood or 8 years of age.

A Swedish questionnaire survey among parents (*N* = 478) suggested that parents could become less concerned about their child over‐ or undereating if they were offered skills training and practical counselling on how to respond effectively to eating behaviours, regardless of the child's weight (Ek et al., 2016).

According to a review by Holmberg Fagerlund, Helseth, Owe, and Glavin (2017), there is limited research on the effect of universal food and feeding counselling involving children under 2 years and their families. At Oslo Metropolitan University, an image‐based communication tool about diet was developed in a previous project named SOMAH (The Research Council of Norway, 2013). This project aimed to facilitate communication about food and feeding practices at CHCs. The target group was immigrant populations with an increased risk of developing type 2 diabetes (Garnweidner, 2013; Holmberg Fagerlund, Helseth, & Glavin, 2019; The Research Council of Norway, 2013). This tool followed the recommendations of the National Nutrition Council in Norway (2011). A selection of the SOMAH images was adjusted and integrated into a communication tool about diet for universal use at the CHC in the present study. A motivational interviewing approach developed by Miller and Rollnick (2013) was integrated into this communication tool to optimize active collaboration about the child's diet between the PHN as counsellor and the collaborating parent.

The aim of the study was to investigate the effect of a communication tool about diet used in a PHN intervention at CHC consultations with parents compared with standard consultations concerning the child's diet at 2 years of age. The study hypothesized that the children in the intervention group would have a higher intake of vegetables, lower intake of saturated fat and lower body mass index (BMI) than those in the control group.

## 3 THE STUDY

### 3.1 Design

**Figure 1:**
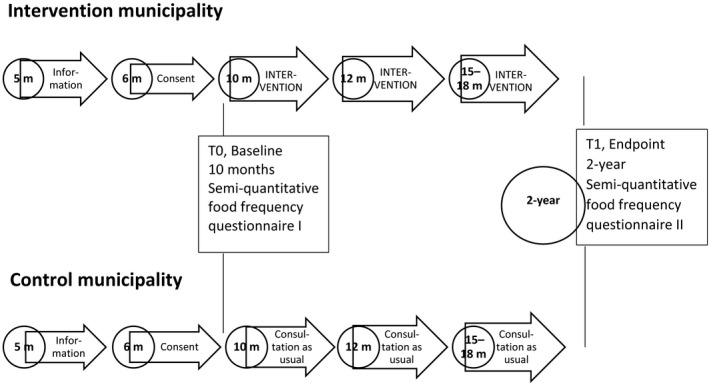
The timeline according to the child's age in the clusters consisting of intervention and control municipalities

The study design was a two‐armed parallel cluster randomized controlled trial (cRCT). Clusters of municipalities were randomly assigned to two groups, intervention municipalities and control municipalities (Figure 1). Parents within the clusters answered a semi‐quantitative food frequency questionnaire (SFFQ) on behalf of their child at baseline (T0) and end point (T1), on average 8–11 months after the end of the intervention. This clinical trial is registered at ClinicalTrials.gov, Identifier: NCT02266953.

### 3.2 Sample

**Figure 2:**
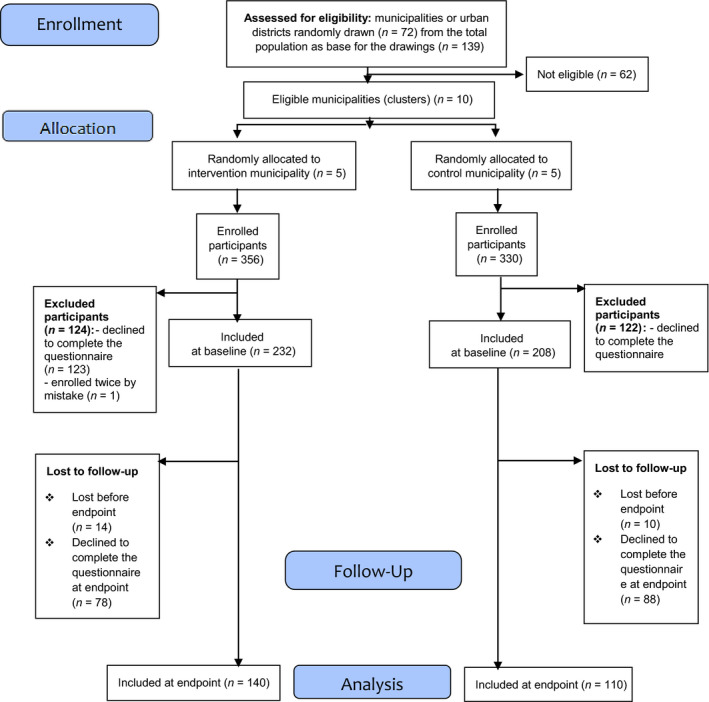
A flow diagram of the participant flow in the cluster randomized controlled trial according to the CONSORT extension for Cluster Trials (Campbell et al., 2012)

In total, five matched pairs of municipalities (clusters) were selected at random by Statistics Norway. The municipalities were matched on the following predefined variables: the number of births in 2012, the number of inhabitants and immigrants in 2013 and the proportion of highly educated inhabitants in 2012. Each cluster in a pair was randomly allocated to a control or an intervention group (Figure 2).

If one cluster in a pair declined to participate, this led to a new draw to obtain a systematic match to the remaining cluster. Thus, obtaining the sample of 10 clusters required contact with 72 municipalities (Figure 2). Twenty‐five of the originally selected municipalities declined to participate in the research project. In addition, 20 of the drawn municipalities could not participate because they had not implemented the healthcare programme at the CHC in a way consistent with the authorities’ regulations. Seventeen were excluded because their CHCs did not practise individual 10‐month consultations.

Municipalities with fewer than 100 births in 2012 and municipalities in the three northernmost counties of Norway were excluded from the draw. Three municipalities were excluded because they had been involved in the development of the intervention. In total, 139 municipalities were available for the sampling.

The municipalities were contacted through their head of the CHC. Oral information about the research project was provided, and if accepted, written information about the project was sent. The study's participants consisted of parents with young children who had consented to participate in the trial before their child reached the age of 10 months. Parents were recruited during their visits at the CHC. The parents received oral and written information about the study from their PHN. The only exclusion criterion was parents with insufficient Norwegian skills to understand the written information about the study. Participants were recruited continuously from 5 January 2015 to 31 January 2017.

### 3.3 Intervention

#### 3.3.1 Control municipalities

The participants and their children in the control municipalities followed the established individual CHC consultations when the child was 10, 12 and 15–18 months old (Norwegian Directorate of Health, 2004). These particular consultations were highlighted because PHNs typically consider counselling about food and feeding practices challenging when the child reaches the age of weaning and gradually begins to eat the same food as the rest of the family (Holmberg Fagerlund et al., 2016). Based on the health authorities’ guidelines, the consultations included an assessment of the child's development and growth. The consultations focused on topics such as breastfeeding and weaning at 10 months, the child's diet, dental health, child–parent interaction, sleep, mobility development, vaccinations and safety concerns. A physician examined the child during the 12‐month consultation (Norwegian Directorate of Health, 2004). The dietary guidelines in effect recommended breastfeeding or formula milk during the first year of life. Gradual adaptation to cow's milk was proposed at 10–12 months of age. The dietary guidelines included suggestions for bread meals and applicable spreads, and some dinner dishes and meal rhythms. Milk types, food texture, dietary composition and vitamin supplements were usually focused on during counselling and in a brochure routinely distributed to parents (Lande & Arsky, 2002; Norwegian Directorate of Health, 2002).

#### 3.3.2 Intervention municipalities

In addition to the content mentioned above, the participants in the intervention municipalities were exposed to the intervention, the PHN’s use of the communication tool about diet. In this intervention, the PHN presented to the parents six or seven printed images per consultation about different nutritional themes related to the child's age and developmental stage (Table 1). The images were A4 size and presented on a flip‐chart stand at the PHN’s desk. To ensure the selection of appropriate image material to be integrated into the communication tool about diet, a feasibility and acceptability test as outlined by Richards (2015) was performed in June–September 2014. Based on these images, parents were invited to discuss relevant themes regarding food and feeding practices, adapted to their family and child.

**Table 1:** An overview of the 20 images in the communication tool about diet

Themes at 10 months:	The image presenting:
1. “Infants learning to feed themselves”	A 1‐year‐old infant sitting in a high chair and using a bib, customized dishes and cutlery, finger food and appropriate food on a plate
2. “The Plate Model” (Camelon et al., 1998)	Proportions of the three food groups: carbohydrates (e.g. potatoes, pasta and bulgur), vegetables and proteins (e.g. meat, fish, beans, peas and eggs) and a spoon of plant‐based oil
3. “Five a Day”	Depicting vegetables and fruit to encourage consumption of at least five portions of them each day
4. Whole grain bread	Depicting healthy alternatives and one example of an unhealthy choice
5. Healthy bread spreads	Depicting healthy alternatives and one example of an unhealthy choice
6. Healthy flavourings of natural yoghurt	Inspiring alternatives of berries and fruit to use as flavouring in yoghurt
7. Natural yoghurt as an alternative to sugary yoghurt	Comparing natural yoghurt without added sugar in relation to the amounts of added sugar in sweet yoghurt types
Themes at 12 months:	
1. “Infants learning to feed themselves”	The same image as at 10 months
2. Milk types	Contents of saturated fat in different types of milk
3. A portion of vegetables or fruit	The child's fist depicting the portion size
4. Whole grain porridge and cereals	Depicting healthy alternatives and one example of an unhealthy choice
5. Healthy flavourings of porridge	Inspiring alternatives of berries and fruit to use as flavouring in porridge
6. Water as an alternative to sugary drinks	Comparing water to the amounts of added sugar in sweetened milk and types of industrially produced juice
Themes at 15–18 months:	
1. “Toddlers learning to feed themselves”	A toddler sitting in a high chair picking food from a plate with a fork
2. “Parent acting as role model”	A mother eating a fruit in front of her toddler
3. Inspiration to choose vegetables	Presentation of 18 types of vegetables
4. Inspiration to choose fruit	Presentation of 18 fruit types
5. Whole grain pasta	Depicting healthy alternatives and one unhealthy choice
6. Whole grain rice	Depicting healthy alternatives and one unhealthy choice
7. Flavoured versus unflavoured milk	Presenting unflavoured milk types in relation to flavoured ones

An aim of the communication tool about diet was to help the family choose an optimal diet for the child, raising the caregiver's awareness of food habits as central to the child's recent and long‐term health. A further aim was to help the family adjust meals to give their child the opportunity to develop skills in eating and get used to different tastes. The authorities’ labelling schemes designating healthy foods and dietary factors according to the authorities’ recommendations were emphasized in the images and during the use of the communication tool about diet.

#### 3.3.3 The time frame in the municipalities

Implementation of the intervention was estimated to take approximately 10 min per consultation, usually regarded as taking between 20 and 30 min in total. Process evaluation was conducted to determine whether the intervention had been delivered as intended and the quantity of what was implemented. According to this, PHNs in the intervention municipalities reported that they used median 35.0 min (min. = 15.0 min, max. = 60.0 min) per featured consultation. Reported median time for performing the intervention was 10.0 min (min. = 2.0 min, max. = 30.0 min). In the control municipalities, the PHNs reported median use of 30.0 min (min. = 20.0 min, max. = 60.0 min) per featured consultation.

#### 3.3.4 Preparations for implementation

Before implementation of the trial, the first author had visited all cooperating CHCs to prepare the PHNs by explaining about the trial and about their tasks. This preparation lasted 2–3 hr on average. In intervention municipalities, a standardized 1‐hr introduction on the use of the communication tool about diet and the corresponding user's manual was included. To minimize likely performance bias and expectation bias related to awareness, information about the study outcomes was withheld during the preparations. This was to enhance objectivity among the cooperating PHNs and participants as described by Polit and Beck (2017).

To accelerate recruitment effort, the PHNs were kept motivated and reminded about this project through monthly contact with their managers by email or telephone. For the same reason, the first author visited all CHCs during the recruitment process.

### 3.4 Data collection

#### 3.4.1 The semi‐quantitative food frequency questionnaires

The SFFQ at baseline, designed to investigate feeding practices of 10‐month‐old infants, was a revised version of a validated and standardized SFFQ developed for a national dietary survey among 12‐month‐old infants in Norway in 2007 (Kristiansen, Lande, Øverby, & Andersen, 2010; Øverby, Kristiansen, Andersen, & Lande, 2009).

The SFFQ at end point was a revised version of a validated and standardized SFFQ developed for a national dietary survey among 2‐year‐old children in Norway during 3 months in 2007 (Kristiansen, Andersen, & Lande, 2009; Kristiansen, Lande, Sexton, & Andersen, 2013).

The revisions and updates in these current SFFQs included new types of formula milk for children, a new type of children's yoghurt replacing an older type, children's porridge containing milk that replaced older types without milk, a soft cheese product that replaced an old type, updated designations of margarines and exclusion of a baby food product no longer on sale. Spinach was removed because it should not be given in large amounts to infants (Norwegian Food Safety Authority, 2011). There was no focus on organic food, and questions about organic food were removed. Questions regarding the weight and length of the child at 6 and 10 months, the mother's use of smokeless tobacco and the mother's country of origin were added in the present SFFQs. Apart from this, the SFFQs consisted of questions described in detail by Kristiansen et al. (2009) and Kristiansen et al. (2010).

At baseline, the parents who had consented to participate received an SFFQ when the child was approximately 8.5 months old. They were asked to complete and return this SFFQ just before the child's 10‐month consultation at the CHC. At end point, the SFFQ was sent to the parents just after the 2‐year consultation at the CHC.

The paper format SFFQs were sent by postal mail accompanied by written information about the survey, a photographic booklet depicting different portion sizes to use when answering the SFFQ and a reply envelope. At baseline and end point, the parents usually received a telephone call to remind them about the questionnaire. Completion of each of the SFFQs was estimated to take about 40 min. Data were collected from 4 March 2015 until 28 June 2018.

#### 3.4.2 Nutrient calculations

Daily intake of energy, nutrients and food groups was computed using a food database in diet calculation software known as *KBS [= KostBeregningsSystemet],* in Norwegian, version 7.3, developed at the Department of Nutrition, University of Oslo. The relevant food database is mainly based on a version of the official Norwegian food composition table (Kristiansen, Laugsand Lillegaard, & Andersen, 2013). The food database AE‐10, based on the official Norwegian food composition table of 2006, was used in the SFFQs (Norwegian Food Safety Authority, 2016).

#### 3.4.3 Age‐ and gender‐related body mass index

Overweight was estimated at the cut‐off point of BMI 25. BMI 25 is equivalent to BMI 18 adapted to the child's age and gender among 2‐year‐old children (Cole, Bellizzi, Flegal, & Dietz, 2000).

### 3.5 Outcomes

#### 3.5.1 Primary outcome

The primary outcome was daily intake of vegetables measured as grams of consumed vegetables per child, based on the completed SFFQs.

#### 3.5.2 Secondary outcomes

The study evaluated several secondary outcomes: the percentage of energy intake (E%) of saturated fatty acids of the total daily energy intake of the child, based on the completed SFFQs; the child's BMI, based on the completed SFFQs; and lastly the number (proportion) of parents who reported a wish to obtain more information about their toddler's diet, based on the completed SFFQs.

### 3.6 Data analysis

#### 3.6.1 Sample size consideration

The study was powered to reveal a predefined change in vegetable intake between the intervention and control groups. According to the literature, children consume on average 50 g vegetables daily at the age of 2 years (Kristiansen et al., 2009). Thus, we expected this daily intake of vegetables to increase by 15 g in the intervention group as compared to the control group. To keep the level of statistical significance at 5% and statistical power of 80% (beta = 20%), we would need 176 children in each group to determine whether the change described above was statistically significant. Attrition was expected, and 300 children were enrolled in each group to make sure our study was sufficiently powered.

#### 3.6.2 Statistical analyses

All statistical analyses were performed using *IBM SPSS Statistics for Windows ®, version 24.0., IBM Corporation®*. Continuous data were described with median and range (data with skewed distribution) or mean and standard deviation (normally distributed data). Categorical data were presented with counts and percentages. Unadjusted differences between intervention and control group were assessed with *t* tests (for continuous normally distributed variables) and the Mann–Whitney–Wilcoxon test if the data were not normally distributed. Chi‐square tests were performed when we tested for association between pairs of categorical data. A correction for multiple testing was performed, and *p*‐values <.01 were considered statistically significant. All analyses were performed according to intention‐to‐treat principles, and no imputation of missing data was performed.

### 3.7 Ethics

All participating parents gave their written informed consent. Participation was voluntary, and the participants could withdraw without giving a reason. All data were treated as confidential. Participant anonymity was guaranteed. Regional Committees for Medical and Health Research Ethics approved the study, reference number: 2014/726. Reporting adhered to the CONSORT extension for Cluster Trials (Campbell, Piaggio, Elbourne, & Altman, 2012).

## 4 RESULTS

Analysis of completers versus non‐completers was conducted at baseline. This revealed comparable groups among the completers and non‐completers of the SFFQ at baseline concerning the background variables, except for fewer married and single mothers and more cohabitant mothers among the completers. This analysis also showed a somewhat higher educational level among mothers and fathers who completed the SFFQ (Table 2). According to process evaluation, the median time for performing the intervention was 10 min in the intervention municipalities.

**Table 2:** Differences between completers and non‐completers at baseline

Variables	Completers (*N* = 250)	Non‐completers (*N* = 190)	*p*‐value*
Mother's age (years), median (range)	31.0 (21–43)	30.0 (18–46)	.27^a^
Missing data	9	14	
Mother's marital status, *N* (%)			
Married	127 (51.2)	108 (57.1)	.04^b^
Cohabitant	117 (47.2)	72 (38.1)	
Not married or cohabitant	4 (1.6)	9 (4.8)	
Missing data	2	1	
Mother's country of origin			
Norway	230 (92.0)	171 (90)	.74^b^
Rest of Europe	14 (5.6)	14 (7.4)	
Outside Europe	6 (2.4)	5 (2.6)	
Mother working outside home or studying^c^			
Yes	81 (32.5)	68 (35.8)	.48^b^
No	168 (67.5)	122 (64.2)	
Missing data	1	–	
Mother smoking			
Yes	14 (5.7)	6 (3.2)	.22^b^
No	230 (94.3)	179 (96.8)	
Missing data	6	5	
Mother using smokeless tobacco			
Yes	15 (6.1)	15 (8.1)	.43^b^
No	229 (93.9)	170 (91.9)	
Missing data	6	5	
Mother's number of children			
1	110 (44.2)	85 (44.7)	.84^b^
2	100 (40.2)	70 (36.8)	
3	31 (12.4)	27 (14.2)	
≥4	8 (3.2)	8 (4.2)	
Missing data	1	–	
Mother's education			
Below upper secondary education	4 (1.6)	6 (3.2)	.04^b^
Upper secondary education^d^	57 (22.9)	61 (32.1)	
Higher education, short	123 (49.4)	71 (37.4)	
Higher education, long	65 (26.1)	52 (27.1)	
Missing data	1	–	
Father's education			
Below upper secondary education	2 (0.8)	10 (5.3)	.04^b^
Upper secondary education^d^	130 (53.1)	91 (48.7)	
Higher education, short	69 (28.2)	51 (27.3)	
Higher education, long	43 (17.6)	33 (17.6)	
Missing data	6	5	
Gestational age of the child at birth (weeks)^d^			
≥38	224 (90.0)	164 (86.3)	.24^b^
<38	25 (10.0)	26 (13.7)	
Missing data	1	–	
Age of the child (months)^c^, mean (*SD*)	9.8 (0.44)	9.8 (0.44)	
Missing data	9	14	.27^a^
Gender of the child^d^			
Female	126 (50.4)	87 (45.8)	.34^b^
Male	124 (49.6)	103 (54.2)	
Child's^e^ birthweight (g), mean (*SD*)	3,526.7 (545.6)	3,558.2 (575.4)	.57^a^
Missing data	7	12	
The child breastfed^c^, *N* (%)			
Yes, currently	130 (52.0)	102 (54.5)	.58^b^
No, but earlier	115 (46.0)	79 (42.2)	
No, never	5 (2.0)	6 (3.2)	
Missing data	–	3	
Parental wish for more information about food for toddlers, *N* (%)			
Yes	138 (55.2)	101 (53.4)	.06^b^
No	102 (40.8)	70 (37.0)	
Do not know	10 (4.0)	18 (9.5)	
Missing data	–	1	
Parents avoid offering applicable food because they are afraid that the child might react with allergy or hypersensitivity			
Yes	81 (33.1)	70 (38.0)	0.28^b^
No	164 (66.9)	114 (62.0)	
Missing data	5	6	

^a^ *t* test for equality of means; equal variances assumed.

^b^ Chi‐square test.

^c^ At the time of completion of the food frequency questionnaire baseline.

^d^ Tertiary vocational education is included.

^e^ The child referred to in the food frequency questionnaire.

* *p*‐values <.05 considered as statistically significant differences between groups.

### 4.1 Participant flow

Two hundred and thirty‐two participants responded to the SFFQ in the intervention municipalities (65% response rate) and 208 in the control municipalities (63% response rate) at baseline (Figure 2). At the end of follow‐up, 140 participants in intervention municipalities (39% response rate) and 110 participants in control municipalities (33% response rate) had completed the SFFQ (Figure 2). Table 3 shows that the distribution of the selected background variables was similar in the intervention and the control municipalities.

**Table 3:** Differences between intervention and control municipalities at baseline

Variables	Intervention municipality (*N* = 232)	Control municipality (*N* = 208)	*p*‐value*
Mother's age (years), median (range)	30 (18–43)	31 (18–46)	.17^a^
Missing data			
Mother's marital status, *N* (%)			
Cohabitant	126 (54.3)	109 (53.2)	.97^b^
Married	99 (42.7)	90 (43.9)	
Single	7 (3.0)	6 (2.9)	
Missing data	–	3	
Mother's country of origin, *N* (%)			
Norway	214 (92.2)	187 (89.9)	.69^b^
Rest of Europe	13 (5.6)	15 (7.2)	
Outside Europe	5 (2.2)	6 (2.9)	
Mother working outside home or studying^c^			
Yes	78 (33.6)	71 (34.3)	.88^b^
No	154 (66.4)	136 (65.7)	
Missing data	–	1	
Mother smoking, *N* (%)			
Yes, daily	4 (1.7)	6 (2.9)	.30^b^
Yes, occasionally	8 (3.4)	3 (1.4)	
No	220 (94.8)	199 (95.7)	
Mother using smokeless tobacco			
Yes, daily	11 (4.7)	11 (5.3)	.83^b^
Yes, occasionally	5 (2.2)	3 (1.4)	
No	216 (93.1)	194 (93.3)	
Mother's number of children			
1	103 (44.4)	92 (44.4)	.44^b^
2	84 (36.2)	86 (41.5)	
3	35 (15.1)	23 (11.1)	
≥4	10 (4.3)	6 (2.9)	
Missing data	–	1	
Mother's education			
Below upper secondary education	5 (2.2)	5 (2.4)	.93^b^
Upper secondary education^d^	62 (26.8)	56 (26.9)	
Higher education, short	105 (45.5)	89 (42.8)	
Higher education, long	59 (25.5)	58 (27.9)	
Missing data	1	–	
Father's education			
Below upper secondary education	7 (3.0)	5 (2.5)	.60^b^
Upper secondary education^d^	125 (54.3)	96 (47.5)	
Higher education, short	58 (25.2)	62 (30.7)	
Higher education, long	38 (16.5)	38 (18.8)	
Missing data	4	7	
Gestational age of the child at birth (weeks)^d^			
≥38	207 (89.2)	181 (87.4)	.56^b^
<38	25 (10.8)	26 (12.6)	
Missing data	–	1	
Age of the child (months)^c^			
Median (range)	9.9 (7.8–11.5)	9.8 (8.2–10.8)	.12^a^
Missing data	13	10	
Gender of the child^e^			
Females	110 (47.4)	103 (49.5)	.66^b^
Males	122 (52.6)	105 (50.5)	
Birthweight (g)			
Median (range)	3,625 (2,480–4,810)	3,240 (2,890–3,900)	.72^a^
Missing data	12	7	
The child gets breast milk^c^, *N*(%)			
Yes	125 (54.1)	107 (51.9)	.52^b^
Not now, but earlier	102 (44.2)	92 (44.7)	
No, has never got	4 (1.7)	7 (3.4)	
Missing data	1	2	
Parental wish for more information about food for infants and toddlers^c^, *N* (%)			
Yes	125 (24.4)	114 (55.1)	.12^b^
No	97 (75.6)	75 (36.2)	
Do not know	10	18 (8.7)	
Missing data	–	1	

^a^ *t* test for equality of means; equal variances assumed.

^b^ Chi‐square test.

^c^ At the time of completion of the food frequency questionnaire baseline.

^d^ Tertiary vocational education is included.

^e^ The child referred to in the food frequency questionnaire.

* *p*‐values <.05 considered as statistically significant differences between groups.

### 4.2 The main outcome

The median age of the included children was 2.2 years in both groups at the time of completion of the SFFQ at end point (Table 4). Our data revealed only small differences in the background characteristics between the groups at 2 years, except for a significantly higher proportion of the mothers in the intervention municipalities who were either married or cohabitants compared to the control group, 98.5% versus 93.6%.

**Table 4:** Differences between intervention and control municipalities at end point

Variables	Intervention municipality (*N* = 140)	Control municipality (*N* = 110)	*p*‐value*
Mother's age (years), median (range)	33 (23–45)	32 (22–45)	.80^a^
Missing data			
Mother's marital status, *N* (%)			
Married or cohabitant	135 (98.5)	103 (93.6)	.04^b^
Not married or cohabitant	2 (1.5)	7 (6.4)	
Missing data	3	–	
Mother's country of origin, *N* (%)			
Norway	126 (92.0)	101 (91.8)	.44^b^
Rest of Europe	9 (6.6)	5 (4.5)	
Outside Europe	2 (1.5)	4 (3.6)	
Missing data	3	‐	
Mother working outside home or studying^c^			
Yes	120 (87.6)	94 (86.2)	.75^b^
No	17 (12.4)	15 (13.8)	
Missing data	3	1	
Mother smoking, *N* (%)			
Yes	10 (7.3)	8 (7.3)	.99^b^
No	127 (92.7)	102 (92.7)	
Missing data	3		
Mother using smokeless tobacco			
Yes	9 (6.6)	7 (6.4)	.95^b^
No	128 (93.4)	103 (93.6)	
Missing data	3	–	
Mother's number of children			
1	44 (32.1)	35 (31.8)	.79^b^
2	65 (47.4)	56 (50.9)	
≥3	28 (20.4)	19 (17.3)	
Missing data	3	–	
Mother's education			
Upper secondary education^d^ and below	27 (19.9)	28 (25.7)	.36^b^
Higher education, short	73 (53.7)	48 (44.0)	
Higher education, long	36 (26.5)	33 (30.3)	
Missing data	4	1	
Father's education			
Upper secondary education^d^ and below	71 (52.2)	57 (52.3)	1.00^b^
Higher education, short	39 (28.7)	31 (28.4)	
Higher education, long	26 (19.1)	21 (19.3)	
Missing data	4	1	
Gestational age of the child at birth (weeks)^d^			
≥38	122 (89.1)	97 (89.9)	0.99^b^
<38	15 (10.9)	12 (11.0)	
Missing data	3	1	
Age of the child (years)^c^			
Median (range)	2.2 (2.0–2.7)	2.2 (1.8–2.8)	.19^a^
Gender of the child^e^			
Female	72 (51.4)	55 (50)	.82^b^
Male	68 (48.6)	55 (50)	
Parental wish for more information about food for toddlers, *N* (%)			
Yes	32 (24.4)	39 (41.1)	<.01^b^
No	99 (75.6)	56 (58.6)	
Missing data	9	15	

^a^ *t* test for equality of means; equal variances assumed.

^b^ Chi‐square test.

^c^ At the time of completion of the food frequency questionnaire baseline.

^d^ Tertiary vocational education is included.

^e^ The child referred to in the food frequency questionnaire.

* *p*‐values <.05 considered as statistically significant differences between groups.

We did not find any statistically significant differences between the intervention group and the control group concerning the main outcome, the mean daily intake of vegetables, 64.5 g versus 68.7 g (Table 5).

**Table 5:** Differences in outcome variables between intervention and control municipalities at end point

Variables	Intervention municipality (*N* = 140)	Control municipality (*N* = 110)	*p*‐value*
Vegetables^d^ (g), mean (*SD*)	64.5 (46.8)	68.7 (83.6)	.54^a^
Saturated fat^d^ (E%), mean (*SD*)	13.0 (2.5)	12.9 (2.6)	.78^a^
Saturated fat^d^, *n* (%)			
<10E%	13 (9.3)	12 (10.9)	.67^b^
≥10E%	127 (90.7)	98 (89.1)	
Child's body mass index (BMI)			
Median (range)	16.4 (13.7–20.43)	16.6 (14.4–19.7)	.19^b^
<18^c^, *n* (%)	106 (88.3)	67 (81.7)	
≥18^c^, *n* (%)	14 (11.7)	15 (18.3)	
Missing data	20	28	
Parental wish for more information about food for toddlers, *N* (%)			
Yes	32 (24.4)	39 (41.1)	<.01^b^
No	99 (75.6)	56 (58.6)	
Missing data	9	15	

^a^ Mann–Whitney–Wilcoxon test.

^b^ Chi‐square test.

^c^ Corresponding to IsoBMI 25 (Cole et al., 2000; Norwegian Directorate of Health, 2010).

^d^ Based on the child's daily intake.

* *p*‐values <.01 considered statistically significant differences between groups.

### 4.3 Secondary outcomes

Our study did not reveal any statistically significant differences between the groups regarding consumption of saturated fat or BMI among children aged 2 years. However, a statistically significant difference was revealed concerning the desire for more information. Fewer of the parents in the intervention municipalities than in the control municipalities reported that they desired more information about food for toddlers, 24.4% versus 41.1% (Table 5).

## 5 DISCUSSION

Our findings showed no statistically significant differences between the two groups on the predefined outcome variables: daily vegetable intake, daily intake of saturated fat and BMI. We do not know if the communication tool about diet was used as intended, to promote a dialogue. The tool might have been used purely for one‐sided information giving, corresponding exclusively to a cognitive parental approach to eating and thus with less impact on their healthy food choices (Marty et al., 2018). Consequently, no effect was attained on the outcomes related to the child's healthy diet. It is not known whether skills training and practical guidance on how to respond effectively to the child's eating behaviours (Ek et al., 2016) were highlighted during the intervention.

The choice of the study's nutritional outcomes, the child's daily intake of vegetables and saturated fat, was seen as reasonable because they are associated with the occurrence of cardiovascular disease in adulthood (Kaikkonen et al., 2013; World Health Organization, 2017). A modest increase in vegetable and fruit intake could have an impact on population health, particularly prevention of deaths from cardiac heart disease (Boeing et al., 2012; Tobias et al., 2006). The current study focused exclusively on vegetables because bitter‐tasting vegetables might usually be harder to accept than fruit initially during the transition to the family's food, due to infants’ innate preference for sweet flavours (Birch & Ventura, 2009). In a previous national dietary survey in 1999 among 2‐year‐old children, the mean daily vegetable intake was 33 g (Kristiansen et al., 2009). Eight years later in 2007, a corresponding national dietary survey among the same age group showed a mean vegetable intake of 54 g/person/day (Kristiansen et al., 2009). Related to the findings one decade ago, the current study showed a general increase in the mean daily intake of vegetables per child to 64.5 g in the intervention and 68.5 g in control municipalities. This positive trend in vegetable intake reflects a similar tendency among the general Norwegian population in the recent years (Norwegian Directorate of Health, 2018). Withholding of information about the outcomes of the trial among participants to prevent performance bias and expectation bias might have been one reason why the intervention did not contribute to the parents changing their child's diet in terms of vegetable intake.

In a systematic Cochrane Collaboration systematic review on “Interventions for increasing fruit and vegetable consumption in children aged 5 years and under,” nine studies were related to children younger than 2 years of age (Hodder et al., 2018). None of these interventions involved the use of an image‐based communication tool in established CHC consultations (Barends, de Vries, Mojet, & de Graaf, 2014; Hetherington et al., 2015; Mennella, Nicklaus, Jagolino, & Yourshaw, 2008; Remy, Issanchou, Chabanet, & Nicklaus, 2013; Roset‐Salla, Ramon‐Cabot, Salabarnada‐Torras, Pera, & Dalmau, 2016; Sullivan & Birch, 1994; Vazir et al., 2013; Verbestel et al., 2014; Watt et al., 2009). According to this Cochrane review, the daily vegetable intake in children younger than 5 years of age increased on average by 3.50 g based on child‐feeding and multicomponent interventions (Hodder et al., 2018). Such a small effect size might limit potential public health benefits from implementing these types of interventions (Hodder et al., 2018).

Our study revealed an intake of ≥10E% of saturated fatty acids among 89%–91% of the 2‐year‐olds and a mean saturated fatty acid intake of on average 13E% in both groups. This is higher than the recommended <10E% and at the same level as in 2007 (Kristiansen et al., 2009). Replacing saturated fatty acids with unsaturated fatty acids could prevent cardiovascular disease (National Nutrition Council in Norway, 2017). To achieve this, it is central to reduce intakes of highly processed fried and nutrient‐poor fast foods and snacks, processed meats and fatty meats. Instead, high‐fibre fruits and vegetables, nuts and seeds, lean meats and fat‐reduced dairy foods should be the core components of children's diets (Te Morenga & Montez, 2017). Our intervention comprising three CHC consultations focused once on the dietary fat content of healthy bread spreads during the 10‐month consultation and once on amounts of fat in milk types during the 12‐month consultation. This intervention might have been too weak to have an impact on any parental food preferences related to saturated fatty acids. To achieve an effect regarding parental selection of less saturated fat in the child's diet, a more assertive intervention has shown positive results. In this longitudinal Finnish study by Kaitosaari et al. (2006), the intervention group parents received individualized dietary counselling twice a year by a physician and a dietitian from when their child was 7 months old. This intervention focused on supporting parents in adopting a healthy low‐saturated‐fat and low‐cholesterol diet for their child. The control group received general health education at the CHC as usual before school age and no in‐depth dietary counselling when the child grew older. This intervention results in children consuming less saturated fat than control children at the age of 9 years (Kaitosaari et al., 2006).

The BMI levels of 11.7% overweight children in the intervention group and 18.3% in the control group correspond to the levels of overweight among 8‐ to 9‐year‐old children in Norway—13% among boys and 17% among girls in 2015 (Norwegian Institute of Public Health, 2018). This reflects the findings of a recent study where similar dietary food patterns were tracked between the age groups of 2 and 8 years (Luque et al., 2018). According to the World Health Organization (2018), increased BMI is a major risk factor for non‐communicable diseases such as cardiovascular diseases, diabetes, musculoskeletal disorders and some cancers. Childhood obesity is associated with a higher risk of obesity, premature death and disability in adulthood. In addition, obese children experience an increased risk of breathing difficulties, fractures and hypertension, early markers of cardiovascular disease, insulin resistance and psychological effects (World Health Organization, 2018). According to a Norwegian population‐based longitudinal study, being overweight or obese at the age of 8 years was associated with an increased BMI throughout infancy and childhood. Hence, interventions to prevent children becoming overweight should start at an early age (Glavin et al., 2014). For instance, increased consumption of vegetables and fruit and regular physical activity could help at the individual level to prevent overweight and obesity (World Health Organization, 2018). Individual‐level interventions targeting healthy eating and physical activity usually have no statistically significant effect on clinical measures of obesity in children (Nigg et al., 2016). The current study's results showing no impact of the individual‐level intervention on the child's BMI were therefore as expected.

Dietary assessments of infants and preschool children appear complicated because their dietary habits often change rapidly (Andersen, Lande, Arsky, & Trygg, 2003). Food served during this age is often not consumed, and gaining an overview of total food intake might be challenging for the parents because of the child's day care (Andersen et al., 2003). Regarding reliability concerns as discussed by Polit and Beck (2017), the accuracy and consistency of information obtained from the completed SFFQs have been central. The chosen age‐specific SFFQs were considered suitable for assessing dietary intake in large groups (Andersen, Lande, Trygg, & Hay, 2004). The SFFQ used among 2‐year‐old children as the basis for the current SFFQ at end point was validated as valuable for measuring average intakes of energy, macronutrients and several food items. Its validity was not influenced by length of the parents’ education or whether the child was attending day care (Andersen et al., 2004).

Our data did not reveal any statistically significant differences at baseline between the groups, so we did not adjust for any possible confounders, and thus, no multiple models were fitted. Further, as the consumption of vegetables was close to zero at baseline, we were not able to model possible changes in this consumption using repeated‐measures methodology.

Use of the communication tool about diet had positive effects on parents in their search for information about food for toddlers. Significantly fewer of the parents desired more information about food for toddlers in the intervention group than in the control group. This might suggest that PHNs in control municipalities spent less time counselling on food and feeding practices because they were not obliged to use any communication tool about diet. PHNs in intervention municipalities reported a median of 5‐min longer consultations totally than the PHNs in control municipalities. However, parental satisfaction with information about dietary concerns regarding their child shows no association with changing their child's dietary habits in a healthier direction. Further studies should focus the PHNs’ evidence‐based knowledge concerning nutrition adapted to infants and children under school age. Moreover, PHNs’ use of the communication tool about diet should be focused on creating a two‐way dialogue adapted to the needs of the parents and effect on healthy dietary choices.

### 5.1 Limitations

Although power analysis was carried out before the study, we did not achieve a large enough sample size based on the preceding calculations because of high attrition among participants who had consented initially. However, we can speculate that even if the calculated sample size had been achieved, it would not have been possible to conclude on any effect of the intervention because the observed differences in the main outcome between the groups were much smaller than anticipated. Based on the literature regarding vegetable intake among 2‐year‐olds (Kristiansen et al., 2009), we initially anticipated that there would be a difference of 15 g/ person/day between the groups. According to a recent Cochrane Collaboration systematic review, child‐feeding interventions appear to increase vegetable intake in children by 3.50 g on average (Hodder et al., 2018), a very small estimate in comparison with our expected achievement. So far, for children there are no guidelines regarding the recommended intake of vegetables (National Institute for Health & Welfare in Finland, 2016).

The usefulness of SFFQs might be questioned because children in this age group eat relatively small amounts of food and their food choices are likely to change. To measure the effect of the communication tool about diet in a relatively short time, behavioural measures or knowledge tests might have been valuable as a supplement to the SFFQ for dietary assessment of the child. However, completion of several questionnaires and tests might have led to even greater attrition due to participant fatigue, according to Polit and Beck (2017).

The fact that the recruitment process lasted for 2 years might have influenced internal validity, because during this time products with a healthy diet focus have been introduced to the market continuously. However, this would have affected both groups concurrently.

The presented sample was similar to the general population in Norway except for the level of education and ethnic background. Only two per cent among mothers and 2%–3% among fathers had *education below upper secondary level* compared to 25.7% of women and 26.7% of men in the general population (Statistics Norway, 2018)*.* Moreover, fewer than 10% of the mothers had an immigrant background compared to 17.7% of immigrants and Norwegian born to immigrant parents in the general population (Statistics Norway, 2019).

The participants having almost exclusively non‐immigrant background and underrepresentation of education below upper secondary level compared to the general Norwegian population might limit the generalizability of our findings. The unknown response rate, based on how many participants were initially invited to participate in the study, also implies a limitation to the generalizability of the results.

## 6 CONCLUSION

Our study did not reveal any differences between the groups on the outcome variables, daily vegetable intake, daily intake of saturated fat or BMI of the child at the age of 2 years. Thus, the PHN using a communication tool about diet in three CHC consultations did not influence parents to choose more vegetables and less saturated fat in their children's diet when compared with the control parents. There were, however, significantly fewer of the parents who desired more information about food for toddlers in the intervention group than in the control group. Our findings indicate that regular nutritional CHC counselling, despite its positive contribution to parents’ information search, will not have any particular impact on the daily intake of specific components in the child's diet such as vegetables and saturated fat.

## CONFLICT OF INTEREST

The authors declare that they have no conflict of interest.

## **AUTHOR** **CONTRIBUTIONS**

BHF, KG, SH, LFA, MCS: Contributions to conception and design, acquisition of data, analysis and interpretation of data, manuscript drafting, critical revision for important intellectual content and final approval of the version to be published. Each author has participated sufficiently in the work to take public responsibility for appropriate portions of the content and agreed to be accountable for all aspects of the work in ensuring that questions related to the accuracy or integrity of any part of the work are appropriately investigated and resolved.

## RESEARCH ETHICS COMMITTEE APPROVAL

The study was approved by the Regional Committees for Medical and Health Research Ethics (REC), Ref.nr. 2014/726.

## References

[nop2498-bib-0001] Andersen, L. F. , Lande, B. , Arsky, G. H. , & Trygg, K. (2003). Validation of a semi‐quantitative food‐frequency questionnaire used among 12‐month‐old Norwegian infants. European Journal of Clinical Nutrition, 57(8), 881–888. 10.1038/sj.ejcn.1601621 12879081

[nop2498-bib-0002] Andersen, L. F. , Lande, B. , Trygg, K. , & Hay, G. (2004). Validation of a semi‐quantitative food‐frequency questionnaire used among 2‐year‐old Norwegian children. Public Health Nutrition, 7(6), 757–764. 15369614 10.1079/phn2004613

[nop2498-bib-0003] Barends, C. , de Vries, J. H. , Mojet, J. , & de Graaf, C. (2014). Effects of starting weaning exclusively with vegetables on vegetable intake at the age of 12 and 23 months. Appetite, 81, 193–199. 10.1016/j.appet.2014.06.023 24973508

[nop2498-bib-0004] Bell, L. K. , Jansen, E. , Mallan, K. , Magarey, A. M. , & Daniels, L. (2018). Poor dietary patterns at 1–5 years of age are related to food neophobia and breastfeeding duration but not age of introduction to solids in a relatively advantaged sample. Eating Behaviors, 31, 28–34. 10.1016/j.eatbeh.2018.06.005 30086453

[nop2498-bib-0005] Birch, L. , & Ventura, A. (2009). Preventing childhood obesity: What works? International Journal of Obesity, 33(Suppl 1), S74–S81. 19363514 10.1038/ijo.2009.22

[nop2498-bib-0006] Boeing, H. , Bechthold, A. , Bub, A. , Ellinger, S. , Haller, D. , Kroke, A. , … Watzl, B. (2012). Critical review: Vegetables and fruit in the prevention of chronic diseases. European Journal of Nutrition, 51(6), 637–663. 10.1007/s00394-012-0380-y 22684631 PMC3419346

[nop2498-bib-0007] Camelon, K. M. , Hådell, K. , Jämsén, P. T. , Ketonen, K. J. , Kohtamäki, H. M. , Mäkimatilla, S. , … Valve, R. H. (1998). The plate model: A visual method of teaching meal planning. Journal of the American Dietetic Association, 98(10), 1155–1158. 10.1016/S0002-8223(98)00267-3 9787722

[nop2498-bib-0008] Campbell, M. K. , Piaggio, G. , Elbourne, D. R. , & Altman, D. G. (2012). Consort 2010 statement: Extension to cluster randomised trials. British Medical Journal, 345, e5661 10.1136/bmj.e5661 22951546

[nop2498-bib-0009] Cole, T. J. , Bellizzi, M. C. , Flegal, K. M. , & Dietz, W. H. (2000). Establishing a standard definition for child overweight and obesity worldwide: International survey. British Medical Journal, 320(7244), 1240 10.1136/bmj.320.7244.1240 10797032 PMC27365

[nop2498-bib-0010] Ek, A. , Sorjonen, K. , Eli, K. , Lindberg, L. , Nyman, J. , Marcus, C. , & Nowicka, P. (2016). Associations between parental concerns about preschoolers' weight and eating and parental feeding practices: Results from analyses of the child eating behavior questionnaire, the child feeding questionnaire and the lifestyle behavior checklist. PLoS ONE, 11(1), e0147257 10.1371/journal.pone.0147257 26799397 PMC4723125

[nop2498-bib-0011] Garnweidner, L. M. (2013). Promoting a healthy diet in antenatal care: Qualitative studies of barriers to nutrition communication among women of different ethnic backgrounds in the Oslo Area. (PhD), University of Oslo, Oslo. Retrieved from https://www.duo.uio.no/bitstream/handle/10852/36083/dravhandling‐garnweidner.pdf?sequence=1&isAllowed=y

[nop2498-bib-0012] Glavin, K. , Roelants, M. , Strand, B. H. , Juliusson, P. B. , Lie, K. K. , Helseth, S. , & Hovengen, R. (2014). Important periods of weight development in childhood: A population‐based longitudinal study. BMC Public Health, 14, 160 10.1186/1471-2458-14-160 24524269 PMC3925776

[nop2498-bib-0013] Hetherington, M. M. , Schwartz, C. , Madrelle, J. , Croden, F. , Nekitsing, C. , Vereijken, C. M. , & Weenen, H. (2015). A step‐by‐step introduction to vegetables at the beginning of complementary feeding. The effects of early and repeated exposure. Appetite, 84, 280–290. 10.1016/j.appet.2014.10.014 25453593

[nop2498-bib-0014] Hobbie, C. , Baker, S. , & Bayerl, C. (2000). Parental understanding of basic infant nutrition: Misinformed feeding choices. Journal of Pediatric Health Care, 14(1), 26–31. 10.1016/S0891-5245(00)70041-1 11141823

[nop2498-bib-0015] Hodder, R. K. , Stacey, F. G. , O'Brien, K. M. , Wyse, R. J. , Clinton‐McHarg, T. , Tzelepis, F. , … Wolfenden, L. (2018). Interventions for increasing fruit and vegetable consumption in children aged five years and under. Cochrane Database Systematic Review, 1, Cd008552 10.1002/14651858.CD008552.pub4 PMC6491117 29365346

[nop2498-bib-0016] Holmberg Fagerlund, B. , Helseth, S. , Andersen, L. F. , Småstuen, M. C. , & Glavin, K. (2018). Parental concerns of allergy or hypersensitivity and the infant's diet. Nursing Open, 10.1002/nop2.195 PMC6279714 30534403

[nop2498-bib-0017] Holmberg Fagerlund, B. , Helseth, S. , & Glavin, K. (2019). Parental experience of counselling about food and feeding practices at the child health centre: A qualitative study. Journal of Clinical Nursing, 28(9‐10), 1653–1663. 10.1111/jocn.14771 30618063

[nop2498-bib-0018] Holmberg Fagerlund, B. , Helseth, S. , Owe, J. , & Glavin, K. (2017). Counselling parents on young children's healthy diet: A modified scoping review. Journal of Clinical Nursing, 26(23–24), 4039–4052. 10.1111/jocn.13892 28543936

[nop2498-bib-0019] Holmberg Fagerlund, B. , Pettersen, K. S. , Terragni, L. , & Glavin, K. (2016). Counseling immigrant parents about food and feeding practices: Public health nurses' experiences. Public Health Nursing, 33(4), 343–350. 10.1111/phn.12248 26813084

[nop2498-bib-0020] Ilmonen, J. , Isolauri, E. , & Laitinen, K. (2012). Nutrition education and counselling practices in mother and child health clinics: Study amongst nurses. Journal of Clinical Nursing, 21(19–20), 2985–2994. 10.1111/j.1365-2702.2012.04232.x 22985324

[nop2498-bib-0021] Kaikkonen, J. E. , Mikkilä, V. , Magnussen, C. G. , Juonala, M. , Viikari, J. S. , & Raitakari, O. T. (2013). Does childhood nutrition influence adult cardiovascular disease risk?–Insights from the Young Finns Study. Annals of Medicine, 45(2), 120–128. 10.3109/07853890.2012.671537 22494087

[nop2498-bib-0022] Kaitosaari, T. , Ronnemaa, T. , Viikari, J. , Raitakari, O. , Arffman, M. , Marniemi, J. , … Simell, O. (2006). Low‐saturated fat dietary counseling starting in infancy improves insulin sensitivity in 9‐year‐old healthy children: The Special Turku Coronary Risk Factor Intervention Project for Children (STRIP) study. Diabetes Care, 29(4), 781–785. 10.2337/diacare.29.04.06.dc05-1523 16567815

[nop2498-bib-0023] Kristiansen, A. L. , Andersen, L. F. , & Lande, B. (2009). [Diet at 2 years; nationwide dietary survey among 2‐year‐old children; 2007]. [Report]. Retrieved from https://helsedirektoratet.no/Lists/Publikasjoner/Attachments/702/Smabarnskost‐2007‐landsomfattende‐kostholdsundersokelse‐blant‐2‐ar‐gamle‐barn‐IS‐1731.pdf

[nop2498-bib-0024] Kristiansen, A. L. , Lande, B. , Øverby, N. C. , & Andersen, L. F. (2010). Factors associated with exclusive breast‐feeding and breast‐feeding in Norway. Public Health Nutrition, 13(12), 2087–2096. 10.1017/S1368980010002156 20707948

[nop2498-bib-0025] Kristiansen, A. L. , Lande, B. , Sexton, J. A. , & Andersen, L. F. (2013a). Dietary patterns among Norwegian 2‐year‐olds in 1999 and in 2007 and associations with child and parent characteristics. British Journal of Nutrition, 110(1), 135–144. 10.1017/s0007114512004643 23192009

[nop2498-bib-0026] Kristiansen, A. L. , Laugsand Lillegaard, I. T. , & Andersen, L. F. (2013b). Effect of changes in a food frequency questionnaire: Comparing data from two national dietary survey instruments among 12‐month‐old infants. BMC Public Health, 13(1), 680 10.1186/1471-2458-13-680 23883290 PMC3724696

[nop2498-bib-0027] Lande, B. , & Arsky, G. H. (2002). *[Food for infants]*. Oslo: Norwegian Directorate of Health Retrieved from https://urn.nb.no/URN:NBN:no‐nb_digibok_2009021600077

[nop2498-bib-0028] Luque, V. , Escribano, J. , Closa‐Monasterolo, R. , Zaragoza‐Jordana, M. , Ferré, N. , Grote, V. , … Ambrosini, G. L. (2018). Unhealthy dietary patterns established in infancy track to mid‐childhood: The EU childhood obesity project. The Journal of Nutrition, 148(5), 752–759. 10.1093/jn/nxy025 29982656

[nop2498-bib-0029] Magnusson, M. B. , Kjellgren, K. I. , & Winkvist, A. (2012). Enabling overweight children to improve their food and exercise habits–school nurses' counselling in multilingual settings. Journal of Clinical Nursing, 21(17–18), 2452–2460. 10.1111/j.1365-2702.2012.04113.x 22686241

[nop2498-bib-0030] Marty, L. , Chambaron, S. , Nicklaus, S. , & Monnery‐Patris, S. (2018). Learned pleasure from eating: An opportunity to promote healthy eating in children? Appetite, 120, 265–274. 10.1016/j.appet.2017.09.006 28890391

[nop2498-bib-0031] Mennella, J. A. , Nicklaus, S. , Jagolino, A. L. , & Yourshaw, L. M. (2008). Variety is the spice of life: Strategies for promoting fruit and vegetable acceptance during infancy. Physiology & Behavior, 94(1), 29–38. 10.1016/j.physbeh.2007.11.014 18222499 PMC2734946

[nop2498-bib-0032] Miller, W. R. , & Rollnick, S. (2013). Motivational interviewing: Helping people change (3rd ed.). New York: Guilford Publications.

[nop2498-bib-0033] National Institute for Health and Welfare in Finland . (2016). Eating together ‐ food recommendations for families with children [electronic book]. Retrieved from http://www.julkari.fi/bitstream/handle/10024/130435/URN_ISBN_978‐952‐302‐626‐1.pdf?sequence=1

[nop2498-bib-0034] National Nutrition Council in Norway (2011). Dietary advice to promote public health and prevent chronic diseases: Methodology and scientific knowledge base. Oslo: The Norwegian Directorate of Health.

[nop2498-bib-0035] National Nutrition Council in Norway . (2017). Dietary guidelines on fats ‐ an update and evaluation of the evidence basis: Executive summary. Retrieved from https://www.helsedirektoratet.no/rapporter/kostrad‐om‐fett‐en‐oppdatering‐og‐vurdering‐avkunnskapsgrunnlaget/Kost%C3%A5d20om%20fett%20%E2%80%93%20En%20oppdatering%20og%20vurdering20av20kunnskapsgrunnlaget.pdf/_/attachment/inline/6dc3e4d8‐3336‐45e3‐9088‐ceaa1beb1278:ed565ff9547d5dd31fed7f46605a9ff3c9d5895f/Kostr%C3%A5d%20om%20fett%20%E2%80%93%20En%20oppdatering20g%20vurdering%20av%20kunnskapsgrunnlaget.pdf

[nop2498-bib-0036] Nigg, C. R. , Ul Anwar, M. M. , Braun, K. , Mercado, J. , Kainoa Fialkowski, M. , Ropeti Areta, A. A. , … Braden, K. W. (2016). A review of promising multicomponent environmental child obesity prevention intervention strategies by the children's healthy living program. Journal of Environmental Health, 79(3), 18–26. 29120137

[nop2498-bib-0037] Norwegian Directorate of Health. (2002). Recommendations for infant nutrition; for health professionals. Retrieved from https://www.nb.no/items/URN:NBN:no‐nb_digibok_2011041208039

[nop2498-bib-0038] Norwegian Directorate of Health. (2004). Guide to the regulations on health promotion and disease prevention in the child health centres and school health services in the municipalities. Retrieved from https://urn.nb.no/URN:NBN:no‐nb_digibok_2011092608169

[nop2498-bib-0039] Norwegian Directorate of Health. (2010). National professional guidelines for weighing and measuring at the child health centres and school health service. Retrieved from https://helsedirektoratet.no/Lists/Publikasjoner/Attachments/236/Nasjonal‐faglig‐retningslinje‐for‐veiing‐ogmaling‐IS‐1736.pdf

[nop2498-bib-0040] Norwegian Directorate of Health. (2016). National professional guidelines for infant nutrition. Retrieved from https://helsedirektoratet.no/Retningslinjer/Spedbarnsern%C3%A6ring.pdf

[nop2498-bib-0041] Norwegian Directorate of Health. (2017). National professional guidelines for health promotion and disease prevention in child health centres, school health services and health centres for young people. Retrieved from https://helsedirektoratet.no/Retningslinjer/Helsestasjons‐%20og%20skolehelsetjenesten.pdf

[nop2498-bib-0042] Norwegian Directorate of Health. (2018). Development of dietary habits in Norway. Retrieved from https://helsedirektoratet.no/Lists/Publikasjoner/Attachments/1479/Utviklingen‐i‐norsk‐kosthold‐2018‐IS‐2759.pdf

[nop2498-bib-0043] Norwegian Food Safety Authority. (2011). Advice for special groups: Infants, (0–12 months). Retrieved from http://www.matportalen.no/rad_til_spesielle_grupper/tema/spedbarn/

[nop2498-bib-0044] Norwegian Food Safety Authority. (2016). The Norwegian food composition table 2006. Retrieved from http://www.matportalen.no/verktoy/the_norwegian_food_composition_table/old_tables

[nop2498-bib-0045] Norwegian Institute of Public Health. (2018). The public health report ‐ health conditions in Norway. Retrieved from https://www.fhi.no/nettpub/hin/

[nop2498-bib-0046] Øverby, N. C. , Kristiansen, A. L. , Andersen, L. F. , & Lande, B. (2009). [Diet at 12 months; nationwide dietary survey among 12‐month‐old children; 2006–2007.] [Report]. Retrieved from https://helsedirektoratet.no/publikasjoner/spedkost‐12‐maneder‐landsomfattende‐kostholdsundersokelse

[nop2498-bib-0047] Polit, D. F. , & Beck, C. T. (2017). Nursing research: Generating and assessing evidence for nursing practice (10th ed.). Philadelphia: Wolters Kluwer.

[nop2498-bib-0048] Rasmussen, M. , Krolner, R. , Klepp, K. I. , Lytle, L. , Brug, J. , Bere, E. , & Due, P. (2006). Determinants of fruit and vegetable consumption among children and adolescents: A review of the literature. Part I: Quantitative studies. International Journal of Behavioral Nutrition and Physical Activity, 3, 22 10.1186/1479-5868-3-22 16904006 PMC1564033

[nop2498-bib-0049] Remy, E. , Issanchou, S. , Chabanet, C. , & Nicklaus, S. (2013). Repeated exposure of infants at complementary feeding to a vegetable puree increases acceptance as effectively as flavor‐flavor learning and more effectively than flavor‐nutrient learning. The Journal of Nutrition, 143(7), 1194–1200. 10.3945/jn.113.175646 23700337

[nop2498-bib-0050] Richards, D. A. (2015). The complex interventions framework In Richards D. A. & Hallberg I. R. (Eds.), Complex interventions in health; An overview of research methods (pp. 1–15). Oxon: Routledge.

[nop2498-bib-0051] Roset‐Salla, M. , Ramon‐Cabot, J. , Salabarnada‐Torras, J. , Pera, G. , & Dalmau, A. (2016). Educational intervention to improve adherence to the Mediterranean diet among parents and their children aged 1–2 years. EniM clinical trial. Public Health Nutrition, 19(6), 1131–1144. 10.1017/s1368980015002219 26258462 PMC10270847

[nop2498-bib-0052] Statistics Norway. (2016). Kommunehelsetenesta; Antall barn som fullført helseundersøkelse ved 4 år. Retrieved from https://www.ssb.no/statbank/table/04685/tableViewLayout1/?rxid=72850577‐f62a‐4dd6‐87d7‐b8d9915c900f

[nop2498-bib-0053] Statistics Norway. (2018). Educational attainment of the population. Retrieved from https://www.ssb.no/en/utdanning/statistikker/utniv

[nop2498-bib-0054] Statistics Norway. (2019). Immigrants and Norwegian‐born to immigrant parents. Retrieved from https://www.ssb.no/en/innvbef

[nop2498-bib-0055] Sullivan, S. A. , & Birch, L. L. (1994). Infant dietary experience and acceptance of solid foods. Pediatrics, 93(2), 271–277. 8121740

[nop2498-bib-0056] Te Morenga, L. , & Montez, J. M. (2017). Health effects of saturated and trans‐fatty acid intake in children and adolescents: Systematic review and meta‐analysis. PLoS ONE, 12(11), e0186672 10.1371/journal.pone.0186672 29149184 PMC5693282

[nop2498-bib-0057] The Research Council of Norway. (2013). Prosjektkatalog 2002–2015: FoU‐satsning Strategiske høgskoleprosjekter ‐ SPH [Strategic Projects ‐ University Colleges]. Oslo: The Research Council of Norway.

[nop2498-bib-0058] Tobias, M. , Turley, M. , Stefanogiannis, N. , Vander Hoorn, S. , Lawes, C. , Mhurchu, C. N. , & Rodgers, A. (2006). Vegetable and fruit intake and mortality from chronic disease in New Zealand. Australian and New Zealand Journal of Public Health, 30(1), 26–31. 10.1111/j.1467-842X.2006.tb00082.x 16502948

[nop2498-bib-0059] Vazir, S. , Engle, P. , Balakrishna, N. , Griffiths, P. L. , Johnson, S. L. , Creed‐Kanashiro, H. , … Bentley, M. E. (2013). Cluster‐randomized trial on complementary and responsive feeding education to caregivers found improved dietary intake, growth and development among rural Indian toddlers. Maternal & Child Nutrition, 9(1), 99–117. 10.1111/j.1740-8709.2012.00413.x 22625182 PMC3434308

[nop2498-bib-0060] Vepsäläinen, H. , Korkalo, L. , Mikkilä, V. , Lehto, R. , Ray, C. , Nissinen, K. , … Erkkola, M. (2018). Dietary patterns and their associations with home food availability among Finnish pre‐school children: A cross‐sectional study. Public Health Nutrition, 21(7), 1232–1242. 10.1017/S1368980017003871 29331168 PMC10261520

[nop2498-bib-0061] Verbestel, V. , De Coen, V. , Van Winckel, M. , Huybrechts, I. , Maes, L. , & De Bourdeaudhuij, I. (2014). Prevention of overweight in children younger than 2 years old: A pilot cluster‐randomized controlled trial. Public Health Nutrition, 17(6), 1384–1392. 10.1017/s1368980013001353 23701835 PMC10282209

[nop2498-bib-0062] Watt, R. G. , Tull, K. I. , Hardy, R. , Wiggins, M. , Kelly, Y. , Molloy, B. , … McGlone, P. (2009). Effectiveness of a social support intervention on infant feeding practices: Randomised controlled trial. Journal of Epidemiology and Community Health, 63(2), 156–162. 10.1136/jech.2008.077115 19141661

[nop2498-bib-0063] World Health Organization. (2017). The top 10 causes of death worldwide. Retrieved from http://www.who.int/mediacentre/factsheets/fs310/en/

[nop2498-bib-0064] World Health Organization. (2018). Obesity and overweight. Retrieved from https://www.wh o.int/en/news‐room/fact‐sheets/detail/obesity‐and‐overweight

